# Retrocecal Appendicitis Post-blunt Abdominal Trauma: A Case Report

**DOI:** 10.7759/cureus.61839

**Published:** 2024-06-06

**Authors:** Juan D Jaramillo, Brayan R Mosquera, Yensy P Botello

**Affiliations:** 1 Surgery, Clínica Colombia, Santiago de Cali, COL

**Keywords:** appendectomy, blunt abdominal trauma, appendicitis, retrocecal appendicitis, case report

## Abstract

Appendicitis, characterized by inflammation and obstruction of the appendiceal lumen, is a common surgical emergency often attributed to various factors. We present the case of an 18-year-old female who developed retrocecal appendicitis following blunt abdominal trauma, a rare occurrence with significant diagnostic challenges. Initial symptoms mimicked upper abdominal trauma, evolving to classic signs of appendicitis within hours. Despite a negative pre-trauma history of abdominal pain, clinical evaluation led to a suspicion of appendicitis. Contrast-enhanced CT scan confirmed the thickening of the cecal appendix, prompting urgent surgical intervention. An open appendectomy revealed a congested retrocecal appendix, supporting the diagnosis. Our case underscores the importance of considering trauma as a potential trigger for appendicitis, especially in the absence of typical pre-trauma symptoms. Diagnostic criteria for post-trauma appendicitis are evolving, and we underscore a comprehensive clinical assessment alongside imaging modalities. While surgical management remains standard, newer approaches like endoscopic retrograde appendicitis therapy warrant exploration. Further research is essential to refine diagnostic and therapeutic strategies for this uncommon presentation, ensuring timely intervention and improved patient outcomes.

## Introduction

Appendicitis is the most common cause of acute abdomen and is considered the most common surgical emergency worldwide. It has a high incidence in young people and is rarer in extreme ages. It is characterized by inflammation and obstruction of the appendiceal lumen due to various causes depending on the age group, including fecaliths, lymphoid hyperplasia, fecal impaction, neoplasms, parasitosis, and trauma, among others [1]. The cecal appendix is considered an intriguing and inconvenient anatomical structure due to its tendency to become inflamed; however, it also serves immunological functions at the gastrointestinal level and as a reservoir of commensal bacteria. It is characterized by a vermiform phenotype that usually measures 6-10 cm in length and 8 mm in diameter with a lumen of 1-3 mm [2], composed of a mucosal layer, submucosa, muscularis propria, and serosa [3].

Appendicitis has been treated using various invasive techniques such as open appendectomy by laparotomy or minimally invasive techniques such as laparoscopy, as well as through more conservative and less invasive approaches such as antibiotic therapy and endoscopic retrograde appendicitis therapy (ERAT) [1,4]. Trauma is considered one of the less frequent etiologies of appendicitis, accounting for only 0.3% of all cases of appendicitis and occurring in 5-15% of cases of blunt abdominal trauma. However, it is not necessarily less important, given the importance of early diagnosis, especially in patients with blunt abdominal trauma due to the numerous differential diagnoses [5]. Below is a case report of an adult patient presenting an unusual case of retrocecal appendicitis following blunt abdominal trauma, highlighting the importance of a better understanding of these less common pathologies and the relevance of early and accurate diagnosis for timely intervention.

## Case presentation

**Figure 1:**
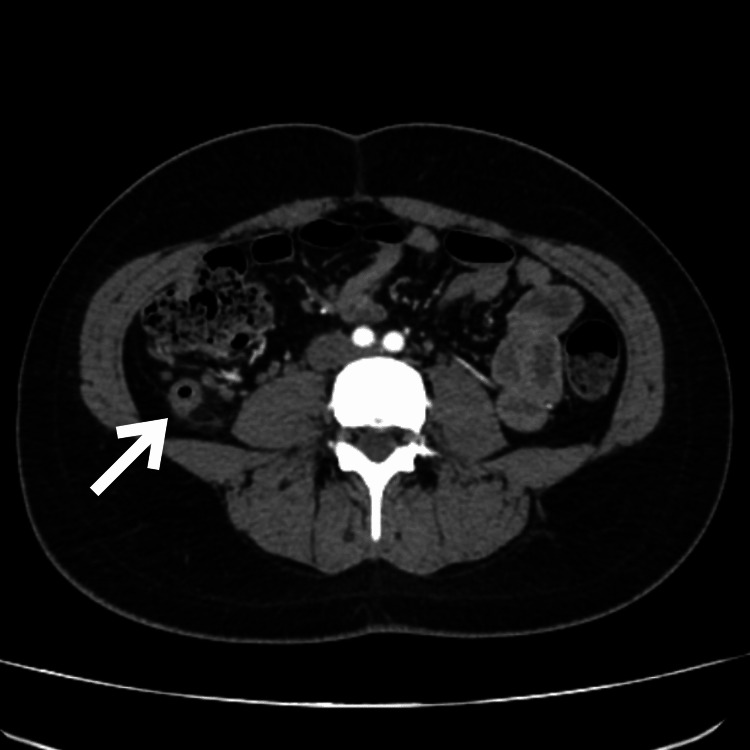
Computed tomography scan showing a dilated appendix, axial view (white arrow).

**Figure 2:**
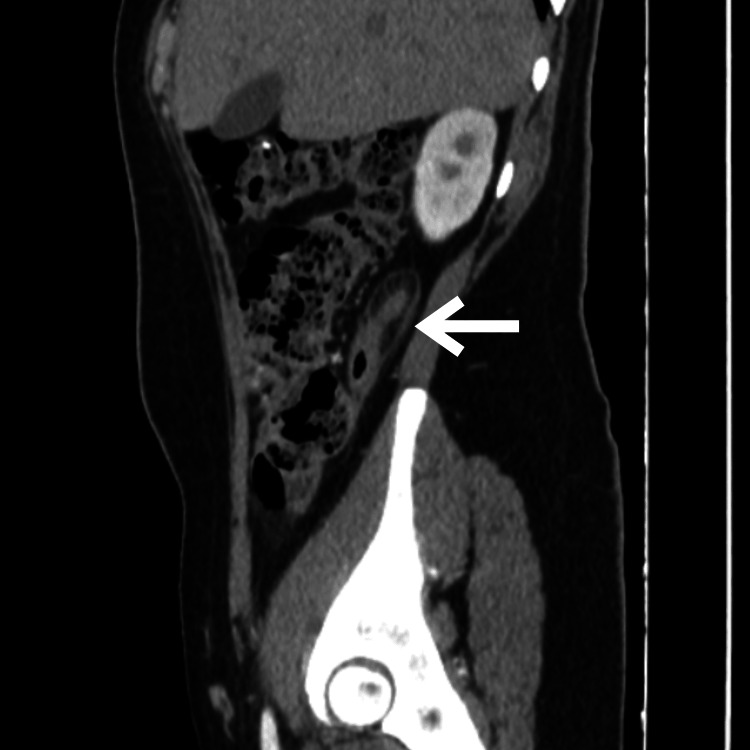
Computed tomography scan showing a dilated appendix, sagittal view (white arrow).

**Figure 3:**
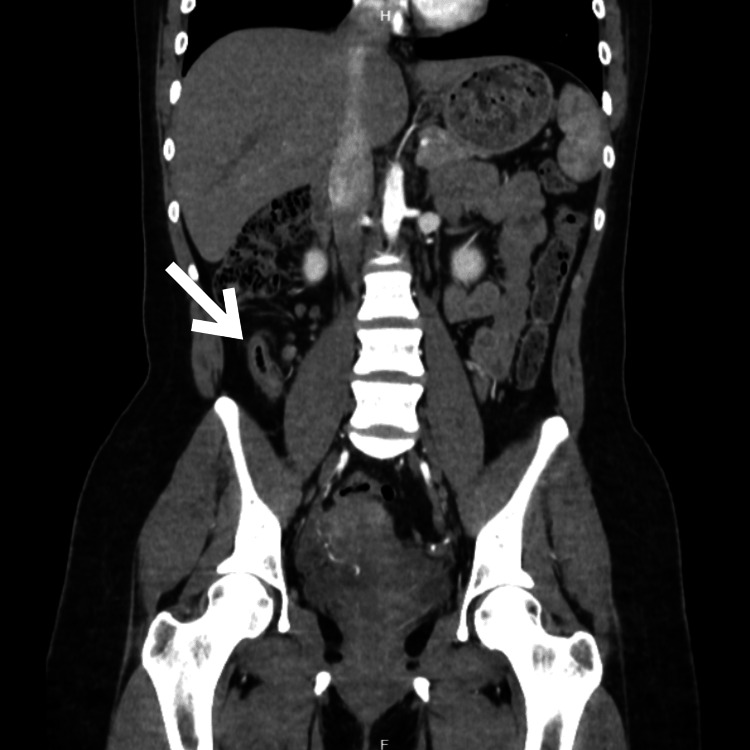
Computed tomography scan showing a dilated appendix, coronal view (white arrow).

An 18-year-old nulliparous female with no significant medical history presented to the emergency department with moderate to severe pain in the right hypochondrial and flank regions immediately following a high-speed car collision while she was a passenger. Upon admission, the review of systems was negative, and the patient denied experiencing any symptoms prior to the car accident. Initially, the pain was localized in the upper abdomen, but after six hours, it migrated to the right iliac fossa and intensified. The patient described the pain as constant and dull, accompanied by anorexia, nausea, and vomiting. Upon checking the vital signs, her temperature was recorded at 36.5°C, pulse rate at 75 beats per minute, blood pressure at 115/75 mm Hg, respiratory rate at 18 breaths per minute, and oxygen saturation at 98% with an FiO2 of 21%. On examination, tenderness was noted in the right iliac fossa, which increased with the psoas maneuver, but rebound tenderness was initially absent. Laboratory findings revealed mild leukocytosis and neutrophilia, normal kidney function, and normal blood clotting times. The Alvarado score for this patient was 7 points, suggesting a likelihood of appendicitis. Therefore, a contrast-enhanced abdominal computed tomography (CT) scan was performed, revealing a thickened cecal appendix measuring 4.6 mm in axial view (Figure 1) and subtle striation of the periappendicular fat better seen in sagittal view (Figure 2); in the coronal view, the presence of gas can be observed in the lumen of the dilated appendix (Figure 3). No other abnormalities were detected.

**Figure 4:**
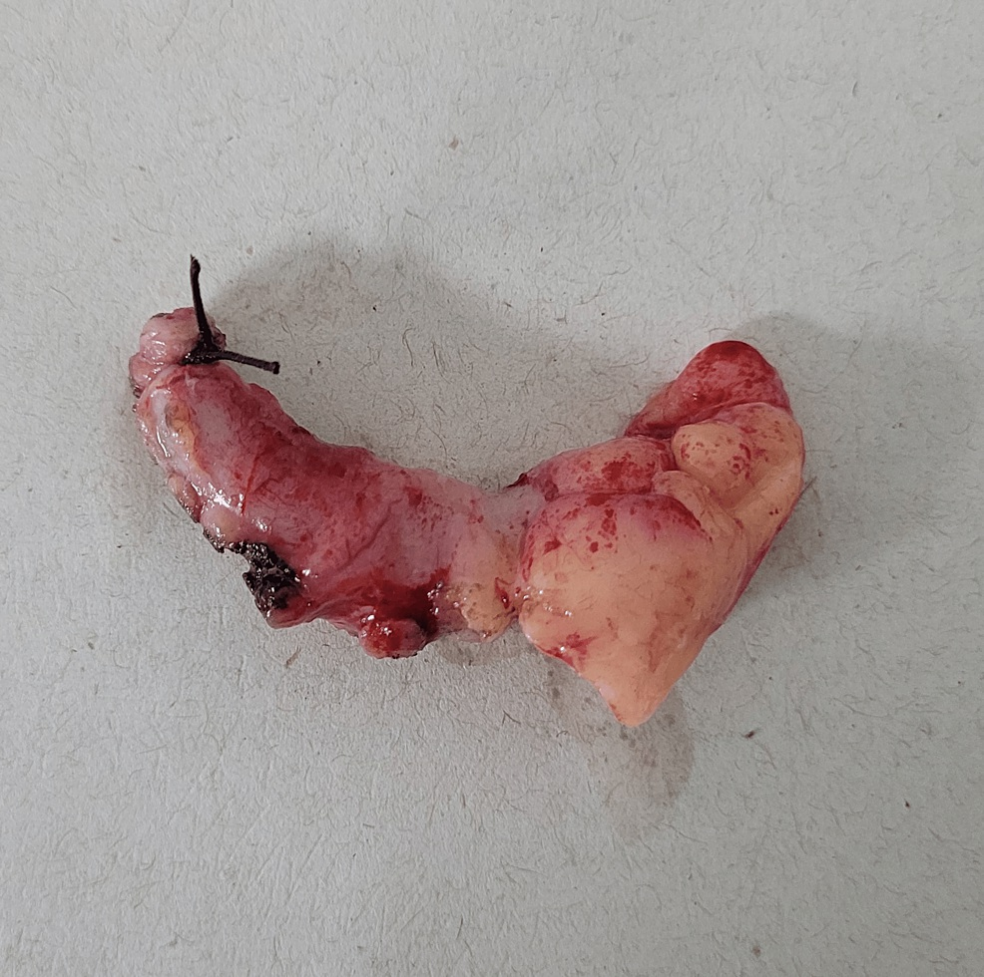
Picture showing inflamed appendix. At the left, the base of the cecal appendix is observed tied with a silk suture.

Twelve hours after her admission, an urgent anesthesia consultation was conducted, without contraindications to surgical management. Since retrocecal appendicitis was suspected based on clinical and imaging findings, and the patient presented with positive rebound tenderness, an open appendectomy was performed using a Rockey-Davis incision. The procedure involved dissection of the planes, Gallaudet fascia, and rectal muscles. Upon exploration of the peritoneal cavity, the surgeon encountered some difficulty but successfully identified the retrocecal appendix. The base of the cecal appendix was dissected, ligated, and cut using the Pouchet technique, resulting in a congested and edematous cecal appendix with minor mucous erosions (Figure 4). Hemostasis was achieved, followed by layered closure of the abdomen without complications. The patient completed a satisfactory postoperative recovery period and was discharged without any further issues after 24 hours.

## Discussion

Blunt abdominal trauma has been associated with intestinal injuries in 5-15% of cases; however, secondary appendicitis following blunt abdominal trauma is an uncommon condition, with an estimated incidence of less than 1% [5,6]. Approximately 8.6% of men and 6.7% of women will experience appendicitis at some point in their lives [7], and in some series, a retrocecal appendicitis incidence as low as 2.5% has been described [8]. Nonetheless, its occurrence after abdominal trauma has been controversial and debated due to its low incidence [9]. However, Fowler conducted the first report in the medical literature and described 48 cases of post-abdominal trauma appendicitis out of a total of 13,496 cases of appendicitis reported in the literature. They described four factors to consider for its diagnosis [10]: (1) absence of abdominal pain prior to trauma, (2) history of violent blunt abdominal trauma with sufficient energy to reach the cecal appendix, (3) presence of typical symptoms and signs of appendicitis, and (4) surgical evidence of appendicitis.

Other authors suggest that given the possible incidental presence of appendicitis, the following four criteria should be considered for post-trauma appendicitis diagnosis [6,9]: (1) absence of abdominal pain prior to trauma, (2) history of direct abdominal wall trauma or acute, unexpected, severe indirect trauma, (3) onset of symptoms between six and 48 hours after trauma, and (4) persistence or progression of symptoms with the surgical confirmation of appendicitis.

The mechanism of trauma associated with these pathologies has been described in traffic accidents, seatbelt injuries, falls from height, and assaults, among others [6]. Various mechanisms have been proposed for the development of acute appendicitis following blunt abdominal trauma, such as the transmission of forces through the organs and tissues of the abdomen to the appendix, resulting in edema, hematomas, obstruction, or injury to the appendix and its vasculature, and even lymphoid hyperplasia, leading to appendiceal lumen obstruction, increased intraluminal pressure, subsequent cecal appendix distension, lymphatic stasis, venous congestion, and subsequent visceral pain and changes secondary to appendix tissue ischemia [5]. Other described mechanisms include increased intra-abdominal pressure, which increases cecal pressure and distends the appendix, leading to altered irrigation and inflammation. Additionally, fecal concretions partially obstructing the appendix may have a greater influence following blunt trauma; mucosal injuries, especially in thin individuals where compression against the iliac bone may occur; and significant forces that can cause appendix rupture due to over-distension and even appendix irritation due to psoas muscle contraction [6,9,10].

In a systematic review conducted between 1991 and 2009, 28 cases with common symptoms described in this case report were found [10]. They also described a clinical presentation similar to that of non-traumatic appendicitis, which is consistent with the criteria proposed by various authors. However, it is noteworthy that in this case, clinical evaluation was crucial in the initial patient management, given the presence of a positive psoas sign suggestive of retrocecal appendicitis, which is clinically important, as patients with post-abdominal trauma appendicitis are more prone to cecal appendix perforation and bacterial migration. Therefore, early diagnosis could prevent complications, especially in retrocecal pathologies that may not manifest early [6,10]. In the patient's case, the trauma occurred directly at the abdominal level, but the pain initially localized in the upper abdomen, and at six hours post-trauma, the pain migrated to the right iliac fossa, along with exacerbation of other signs suggestive of appendicitis.

While many authors recommend imaging studies in patients with suspected acute appendicitis, abdominal ultrasound or even extended focused assessment with sonography in trauma (eFAST) can be a good first choice, but abdominal CT scan has proven to be very useful as a confirmatory study in blunt abdominal trauma with signs suggestive of appendicitis; it also allows the evaluation of other abdominal structures, which is relevant given the numerous possible differential diagnoses in these types of traumas [11].

Routine laboratory tests such as hematologic and biochemical studies should not be considered useful tools on their own; in fact, they are often considered unuseful, as elevation of acute-phase reactants is common but sometimes absent, and it does not rule out appendicitis [11].

In this patient's case report, the first symptom was abdominal pain, which is invariably described in all cases, although not always manifested at the level of the right iliac fossa and more commonly evidenced as diffuse pain and, to a lesser extent, at the level of the upper abdomen [5,11]. It is evident that all these theories focused on mucosal edema and appendix lumen obstruction secondary to trauma; therefore, the description of the type of trauma and its kinetics are particularly important, as well as a complete physical examination [10]. Considering that several authors indicate that a wide range of differential diagnoses may be considered, these types of diagnoses are usually considered as a last resort; however, it is important to also consider them in patients with high-energy blunt abdominal trauma, especially within the first 72 hours post-trauma [6,11]. Currently, the Alvarado score or other scales for evaluating post-blunt abdominal trauma appendicitis have not been validated. The approach to patients with appendicitis may influence treatment choice, which can be operative or non-operative with antibiotic therapy implementation in specific and selected cases of uncomplicated acute appendicitis, with the advantage of avoiding anesthesia and surgery but with a higher risk of recurrence rates and increased risk of developing complicated appendicitis with increased intraoperative and postoperative morbidity [1,12]. Depending on the patient, minimally invasive procedures with lower morbidity and mortality, such as laparoscopy or ERAT, may be chosen, with the latter being a newer treatment described since 2012 by Liu and subsequently by other authors, with an efficacy between 92% and 97% and lower morbidity and mortality [1,4]. However, there are no records of ERAT in patients with post-blunt abdominal trauma appendicitis, and in many cases, concerns arise regarding the risk of hollow viscera injury and its derived complications; therefore, operative management is usually opted for in these patients [1,11]. In the patient's case report, open appendectomy was chosen, which is considered a standard treatment for non-traumatic acute appendicitis and the most described procedure in post-blunt abdominal trauma appendicitis treatment, as well as laparotomy and, to a lesser extent, laparoscopy [11], which in some case reports has required conversion to laparotomy [6]. However, more studies are needed to evaluate the efficacy of these surgical procedures in post-blunt abdominal trauma appendicitis management. The morbidity and mortality of patients with post-blunt abdominal trauma appendicitis vary depending on the procedure, etiology, patient, and their history, among others [1]. In the patient's case report, since it was uncomplicated appendicitis post-blunt abdominal trauma, she could be discharged 24 hours after admission; however, Toumi et al. described in a systematic literature review an average of 11 days of hospital stay and 0% mortality in the described case reports [13]. No other reports on morbidity and mortality in these types of patients were found upon literature review, so further studies are needed to better characterize patients with post-blunt abdominal trauma appendicitis.

## Conclusions

Although trauma is one of the less frequent causes of appendicitis, physicians must keep this etiology in mind to ensure their differential diagnosis becomes more effective and prevent underestimating potential acute abdomen pathologies. While there are numerous algorithms, scores, and laboratory and imaging diagnostic tools available, it is important to emphasize the significance of obtaining a thorough clinical history, conducting semiotics, and performing a comprehensive clinical evaluation as the first approach to identifying and guiding patient diagnosis and treatment.

Further studies are needed to determine and standardize the necessity and indication of the various diagnostic studies available to date. Additionally, given the gap in knowledge, more research are required to determine the effectiveness of different medical and surgical approaches and to better characterize this population of interest. This case serves as a reminder that physicians must consider different etiologies and differential diagnoses in certain special scenarios and conduct a comprehensive clinical evaluation.
